# Supplementary material for: Programmed Cell Death Reversal: Polyamines, Effectors of the U-Turn from the Program of Death in Helianthus tuberosus L
Source: Int J Mol Sci. 2024 May 15;25(10):5386. doi: 10.3390/ijms25105386 (PMC11121942; doi:10.3390/ijms25105386)
Supplement: Supplementary file 1 [file ijms-25-05386-s001.zip › ijms-2821172-supplementary.pdf]

Supplementary Material: History of polyamine research and their properties in plants

1. Foreword

This supplemental part reports the general characteristics and the main steps in the discovery of the polyamines (PAs) in plants not reported in the previous manuscript to not interrupt it. Here the data are taken from general PA literature, also adding complementary data on *Helianthus t.* and other plants. This part has also the target to show a short history of the development of PA research and the scientific logic applied by these studies in the Polyamine Laboratory of the Bologna University since 1965 until now.
The figures are cited with an "S" before the number to distinguish them from those of the main manuscript.

2. Polyamine history in plants

As reported in the main manuscript, in plants the PAs role as growth factor was discovered in *Helianthus t.* in 1965 [1]. Since that time, a flowering of discoveries developed. Here we report the milestones of the research conducted not only in our lab, but also in other labs. At first PAs were studied in relation to their effect on nucleic acids, promoting protein synthesis [2], as at that time data at molecular level were reported on interactions with DNA and different RNAs. Successively, starting from the years 1985, PA binding with proteins were observed in *Helianthus t.* [3] and then this field of research was enlarged due to the possible regulations induced in protein functionality. Also PA metabolism and transport were object of several innovative researches, conducted especially on *Helianthus t.*. The multiple PA functions were confirmed by many other research summarized in some reviews [4,5,6]. More recently, in the years 1987-1989, an enzyme, transglutaminase, was identified and found to catalyze the conjugation of PAs to specific plant proteins [7, 8] and a new line of research was developed, as reported by the papers [9-13]. Transglutaminase research opened a new panorama on protein covalently bound PAs, previously neglected. In many cases this is the molecular condition by which PAs exert their multiple roles in the cell. In fact, PAs can bind to molecules, especially macromolecules, regulating their properties [5, 14, 15, 16, 7, 17, 18, 19, 20, 21, 22, 23, 24, 25, 9, 11, 26, 27, 10, 28, 13, 29, 30, 31, 32].
The plant organisms object of all these researches were mainly *Helianthus t.*, either dormant or activated *in vitro*, the pollen and its germination in different Rosaceae fruit trees, the flowers of *Nicotiana sp.* and the unicellular alga *Dunaliella salina.* A tenth of other Tracheophyta plants, a virus and a bacterium a were less frequently studied. The main biological events considered were dormancy, *in vitro* or *in vivo* growth, flowering, pollen development, photosynthesis, cell cycle, programmed cell death.

3. Polyamine molecules

The biogenic polyamines, common to practically all living organisms, play widespread and vital roles evolutionary ancient. The crystals of PAs were observed in 1678 by Antony van Leeuwenhoek [33] in human semen, but were identified only in the last century [34].
Aliphatic low-molecular-weight polyamines are polycationic bases formed by a linear carbon hydrophobic backbone, having two terminals highly protonated aminic groups and eventually one or two internal iminic ones, thus being electronically reactive in the cell.
The most widespread PAs are the diamine putrescine (Put) the triamine spermidine (Spd) and the tetramine spermine (Spm) (Figure S1A) [33, 35]. Their backbone is flexible, see an example of Spd in Figure S1B, which have the capacity to adapt to the form of other molecules to which might be linked [35, 36]. Other aliphatic natural PAs exist, among which thermospermine and cadaverine, the last is the precursor of some alkaloids [33, 37, 38, 39].

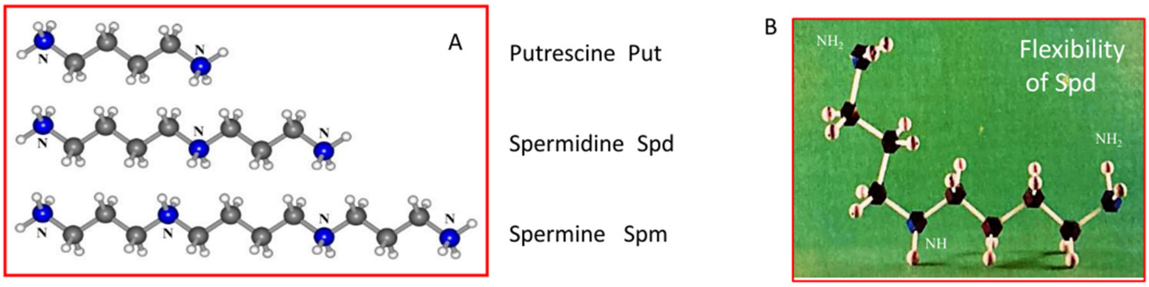

Figure S1. Structure of PAs
A. Structure of the three main aliphatic polyamines: Putrescine, Spermidine and Spermine. The N atoms of the terminal aminic or internal iminic groups are in blue color.
B. Flexibility of the aliphatic backbone, example of Spd.

4. Localization, metabolism, transport and roles of polyamines

It is almost a universal rule that all living organisms require polyamines to keep viability [4]; however, the precise biochemical functions of PAs as cytoprotective molecules are still an incompletely resolved topic. These molecules have the property to be transferred easily and rapidly among the different parts of the cell as well as among different cells. Part of these molecules can become bound and thus fixed to cell macromolecules and cell structures.

The **PAs localization** in the cell depends on the balance among biosynthesis and degradation but also by transport (efflux and uptake) [40, 41]. PAs are in fact actively transported among cellular organelles/compartments and the extracellular space [42, 43]. PA import was evidenced for the first time, in protoplasts and vacuoles [44, 45] moreover, the PA uptake was detected in mitochondria of *Helianthus t.* as well as in chloroplasts [46, 47]. The uptake is concentration-dependent, stimulated by Ca2+, energy-dependent, auxin-regulated and protein-mediated [48, 49].

PAs are transported in the entire plant also bidirectionally at long distance via xylem and phloem [50, 51, 52] with a different distribution [47, 53, 14].

In addition, being transported among different compartments and entire cell, PAs are metabolized. The **PA metabolism** is rather complex, showing that PAs can be actively interconverted. A precise optimum of PAs is necessary for each single function, and it must be finely regulated by these mechanisms.

The enzymes of the PAs metabolism are frequently compartmentalized [41, 54]. Put is the precursor of Spd directly from ornithine by the ornithine decarboxylase (ODC) [55] or indirectly from arginine by arginine decarboxylase (ADC) pathway. Spd derives from Put and Spm derives from Spd by the respective synthases. Glutamine, deriving from Krebs cycle, can act as precursor of PAs [56] .

Spd and Spm are retro-converted to Put by polyamine oxidase (PAO) in the peroxisomes and apoplast, with the generation of H_2O_2, DAP and other products, participating in plant defense and plant-bacteria communication. Diamino oxidase (DAO) oxidizes Put and also Spd. It has been shown that PAs can function as sole nitrogen source for *Helianthus t. in vitro* [56] showing that they can be efficiently converted in all primary metabolites by the complex PA interconversion. Moreover, PAs might exert different roles also when are converted into secondary metabolites [57], among which cytotoxic compounds, scavenging radicals, alkaloids [37], H_2O_2 which affects pathogen growth, induces cell-wall reinforcement and stomatal closure.

The metabolism of PA is subjected to a circadian rhythm, which strongly influence it [58]. In addition, stresses also have a very relevant effect on the metabolic pathways, as largely reported in literature, [59,60] and Spm improves stresses tolerance [61, 62].
As a consequence, not only of their transport but also of their metabolism, PAs in fact have been found compartmentalized practically in all cell organelles or structures as shown in (Figure S2A) where PAs have roles related to the function of the different parts. Their functions have effects at subcellular, cellular and organismic levels (Figure S2B,C,D) [14, 55, 63, 64].

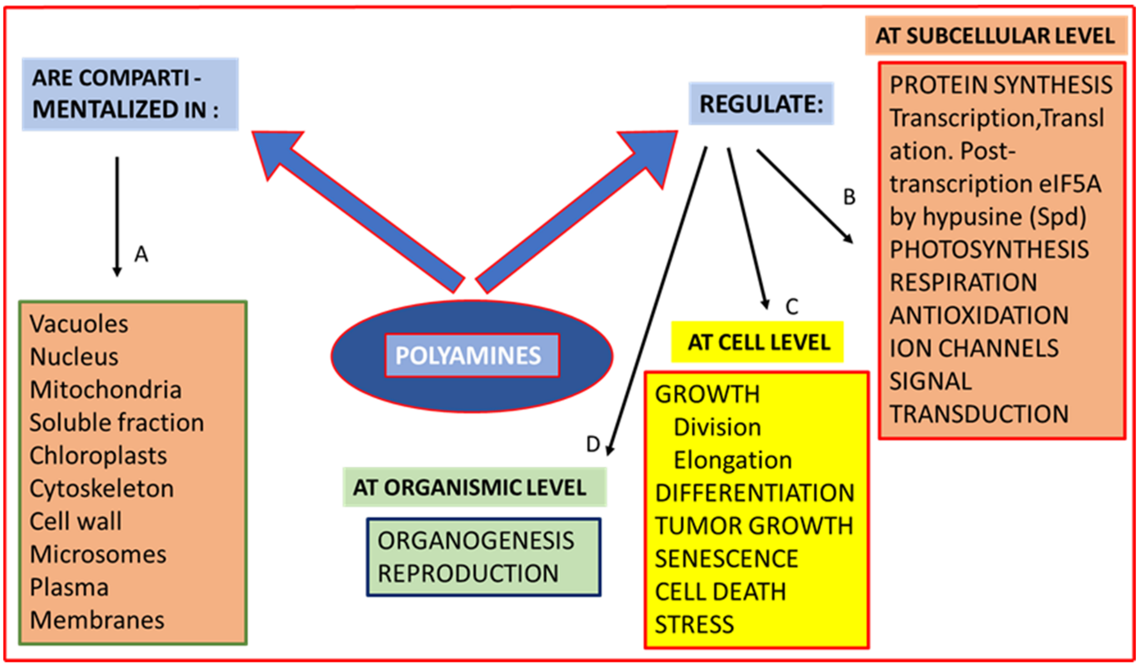

Figure S2. Localization and regulation of different functions of PA.
A. Compartmentalization of PAs in different structures or organelles of the cell.
B. - D. PA regulatory role at: B. subcellular; C. cellular; D. organismic events.

In the different compartments PAs might be involved in photosynthesis, respiration, protein synthesis, signal transduction, regulation of ion channels, membrane potential, electrolyte balance, in cell homeostasis.
About the role of PAs in cellular organelles, the chloroplast is probably the most studied organelle for the peculiar PA role in the more specific and energetically relevant function in plants, namely photosynthesis (Figure S2A,B). The presence of PAs in chloroplasts was firstly reported in the alga *Euglena g*. in 1974 [65] and then PAs were found in all plants examined. PAs regulate structure and functioning of the photosynthetic apparatus, being present in thylakoid and stroma compartments. The conversion of pro-plastids into chloroplasts, observed in *Helianthus t.* during *in vitro* culture in the light, is stimulated by the presence of PAs [66].
As a consequence of their presence in various organelles, PAs have many regulative roles at cell level (Figure S2C) and influences many fundamental cell processes, such as cell division, elongation, differentiation, senescence/PCD, stress- and external stimuli-induced, homeostatic adjustments etc. [43, 67]. These distributions in the plant cell have consequences also on the organ differentiation (Figure S2D), such as embryogenesis, organogenesis of roots, leaves, flowers, tubers and fruits [52]. PAs are also critical in

reproduction, self-incompatibility from pollen development to fertilization (Figure S2D) [12, 68].
The single PA has different roles in plant cell life. PAs act as pro-survival molecules, as rejuvenation factors [**69**], whereas in other conditions they accelerate cell death [**70**]. Put frequently contrasts Spd and Spm, emphasizing that individual biogenic amines have defined action and they differentially affect growth and development [71].
Usually, PAs are present at high concentrations in the growing organs [72], and tumors represent an important model. In animals a correlation between the level of PAs and tumor growth velocity was established. The role of PAs in cancer, and also in many other pathologies, assumed great relevance in mammal pathophysiology [72, 73].
Similarly to other growth substances, the relationship between growth rate and concentration of PAs is represented by a Gaussian curve: PA depletion or excessive accumulation may both have inhibitory effects. DNA synthesis and cell viability can be compromised by extreme levels of PAs; moreover, an increase in PA degradation involves the generation of aldehydes, some of them highly toxic.

5. Free and bound PAs.
PAs are present in the cells either in free or in bound forms, namely linked to other molecules of which could modify either their chemical charge or structure [5, 74].
The free ones (Figure S3A) could exert their roles as long polycations, being mostly charged at the physiological cell pH. Free PAs are easily metabolized, transported, exchanged and possibly could have an osmotic role and contribute to the activation of gene expression.

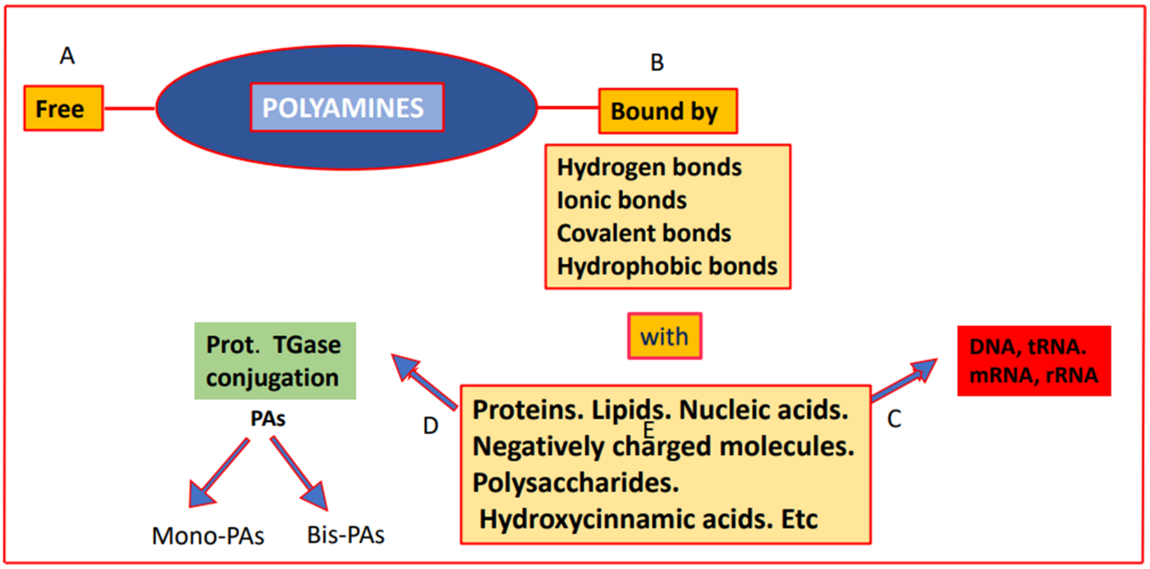

Figure S3. PAs in free and bound forms
A. Free PAs.
B. Bound PAs by different types of bonds.
C. Binding with nucleic acids.
D. Binding with proteins by means of transglutaminase (TGase). The PAs binding gives rise to: mono- (glutamyl)-PAs or, by a second transamidase reaction, to bis- (glutamyl)-PAs [**75**].

Mainly free polyamines have been studied and reviewed [71, 76, 77]. Several activities, attributed to free PAs, can instead be performed by PAs bound in a not stable form.
PAs in both free and bound forms are present in *Helianthus t.* tuber and in primary meristem of sprout apices [7,78].
PAs have high electrostatic affinity for negatively charged molecules and become bound. This term comprises PAs tightly-bound and non-tightly-bound ones.
As shown in Figure S3B, there are different types of bindings: hydrogen, ionic, covalent and hydrophobic. When aminic and iminic groups interact by ionic and hydrogen linkages with negatively charged groups of several molecules are easily reversible. Other instead are stable, like for example the covalent ones that can be disrupted only by high acid concentration hydrolysis in hot condition [16]. Conjugated PAs represent only a fraction of the total bound entities and should not be construed as an alternative definition of 'free'. Hydrophobic interactions primarily involve the lipids of membranes, proteins, nucleic acids, other lipids, cell walls, and so on, and they are reversible [79].
In addition, the PAs, when extracted, are classified in two fractions, depending on their solubility in perchloric or trichloric acids (PCA or TCA): PCA- (or TCA-) soluble and insoluble, depending on the molecular weight of their partners (below or higher than 5000 Da).
PAs can be regarded as charge donors or bridge builders. In fact, they can modify biomolecules, as nucleic acids, producing a charge effect and interacting with helical structures [77] (Figure S3C).
For example, rRNA is active in amino acid incorporation, only when it formed a complex with PA, like in *Helianthus t.* tuber cells, described in the main manuscript [80].
Another example of PA binding, a covalent one, might occur with proteins (Figure S3D), but this is catalyzed by an enzyme, transglutaminase, below described. PAs can form only a single binding (mono-PAs) cationizing the protein, or bis-PAs forming a bridge between two proteins.
Other linkages can occur with lipids, polysaccharides, hydroxycinnamic acids, etc.

5. 1. Types of PA interactions
5. 1. 2. Non-tightly-bound interactions
Important examples of non-covalent linkages of PAs are those with nucleic acids.
Due to their linkages, PAs interfere with transcription [81]. The interaction between PAs and DNA has been observed also *in vitro* and *in vivo* in *Helianthus t.* [17, 18]. PAs play a molecular stabilizing role, either by interacting with the double helix or also by the covalent binding to histones [82]. Moreover, PAs are also non-tightly-bound bound to tRNA, rRNA, 5SRNA, mRNA. PAs exert several roles in the protein synthesis, acting at different levels, chromatin organization, transcription, translation [83], but PAs may also interact post-translationally with proteins. PAs interact with the membranes [5] important for the transmission of receptor-mediated signals [85].
The binding of PAs to proteins could be non-covalent or covalent. The first one is not stable. PAs have been found not covalently bound also to hemicelluloses, pectins, cellulose and lignin [86, 87]. PAs covalently bound to proteins are widespread: as an example, PAs are accumulated in the cell wall, where they interact with polysaccharides as well as by covalent linkages with proteins.

5.1.3. Tightly-bound interactions
In addition to non-tightly bound PA interactions, a large percentage of PAs can also be conjugated to other low-molecular-weight compounds [88, 89, 90], like hydroxycinnamic acids, involved in the organization of the cell wall and associated to fertility [91, 92].
PAs are also bound with high molecular mass molecules, namely proteins (Figure S3D). A widespread family of enzymes are responsible for this binding: transglutaminases (TGases) which frequently catalyzes high-order molecular complexes by PAs bridges. These bridges might have a structural role stabilizing protein structure by their flexible alkyl chains (Figure S1B), which can adapt to various conformations upon binding. Frequently high-order molecular complexes by PAs bridges are catalyzed, having a structural role in the stabilization of protein structure.

In plants TGases were found to catalyse the covalent binding of PAs to some glutamyl-residues of specific proteins. PAs can form only a single binding, mono-PAs, cationising the protein, or bis-PAs forming a bridge between two proteins if the second terminal amino group of the mono conjugated PA, is linked. When many of these bindings are formed, a protein net can be produced, eventually of high mol mass, giving rise to large structures, for example cytoskeletal ones.
The inter- or intra-protein PA bridges are of different length according to the kind of PAs and reduce the repulsive forces between negatively charged components, leading their aggregation [79, 84, 93].
In the 1980s, the presence of TGase-like activity was first detected and biochemically characterized in the sprout apices of *Helianthus tuberosus* [7], and subsequently observed during its tuber cell cycle [22] and in other plants [94]. This activity was later confirmed in *Arabidopsis thaliana* and *Zea mays* [95, 24]. Multiple forms of these enzymes were found in all tracheophyte organs tested, such as primary meristems of the tuber sprout, tubers, roots, leaves, flowers, activated tuber parenchyma, seeds, pollens, in different organelles and in algae [9, 96]. These TGases in plants have been reviewed [9, 11, 13, 26, 97].
The identification of the substrates allows to clarify the role of some of these TGases. In fact, some protein substrates are typical of certain plant cell organelle. The main TGase substrates were detected in the chloroplasts, also of *Helianthus t.* leaves and their isolated compartments, like Rubisco in the stroma and in several components of Light Harvesting Complexes of thylakoids, modifying their conformation in a light-dependent way. This influences photosynthesis and photoprotection, favoring growth and delaying senescence [24, 27, 66, 89, 98, 99, 100, 101, 102]. Since PAs act as radical scavengers, resistance to abiotic stress is conferred on the photosynthetic apparatus, affecting the photosynthetic efficiency against various stress factors [10, 28, 98]. In addition, PAs could react with atmospheric CO2 [103]. Among other cell substrates of TGase, the cytoskeleton proteins, tubulin and actin, are affected in their cytoplasmic movements with effects on mitosis, organelle position etc. [30, 31].
Other plant substrates are those of cell walls and membranes: their presence and role in regulating permeability are revealed by their rigidification during senescence and accelerating cell wall differentiation [32, 69]. An extracellular form is necessary for the growth of pollen tube possibly by rigidifying it [104,105]. Moreover, the externalised pollen TGase could be one of the mediators of pollen allergenicity [106].

Conclusion

The narrative of PAs seems infinite, making it particularly challenging to provide a comprehensive overview that elucidates all the principal roles of PAs. This narrative span various phases and objectives of research. Numerous reviews are referenced, delving into details that are merely summarized in this report. The purpose here is to offer some fundamental information to facilitate the understanding of the historical research journey concerning PCD reversal in *Helianthus t.*.

REFERENCES

1. Bertossi, F.; Bagni, N.; Moruzzi, G.; Caldarera, C.M. Spermine as a New Growth-Promoting Substance for *Helianthus tuberosus* (Jerusalem Artichoke) *In vitro*. Experientia 1965, 21, 80–81, doi:10.1007/BF02144752.
2. Serafini-Fracassini, D.S.; Bagni, N.; Cionini, P.G.; Bennici, A. Polyamines and Nucleic Acids during the First Cell Cycle of *Helianthus tuberosus* Tissue after the Dormancy Break. Planta 1980, 148, 332–337, doi:10.1007/BF00388120.
3. Serafini-Fracassini, D.; Mossetti, U. Free and Bound Polyamines in Different Physiological Stages of *Helianthus tuberosus* Tuber. In Recent Progress in Polyamine Research; Selmeci L., Brosnan M.E., Seiler N, 1985; pp. 551–560.
4. Bagni, N.; Serafini-Fracassini, D. Involvement of Polyamines in the Mechanism of Break of Dormancy in *Helianthus tuberosus*. Bull. Soc. Bot. Fr. Actual. Bot. 1985, 132, 119–125, doi:10.1080/01811789.1985.10826718
5. Bagni, N.; Tassoni, A. Biosynthesis, Oxidation and Conjugation of Aliphatic Polyamines in Higher Plants. Amino Acids 2001, 20, 301–317, doi:10.1007/s007260170046.
6. Tassoni, A.; Bagni, N.; Ferri, M.; Franceschetti, M.; Khomutov, A.; Marques, M.P.; Fiuza, S.M.; Simonian, A.R.; Serafini-Fracassini, D. *Helianthus tuberosus* and Polyamine Research: Past and Recent Applications of a Classical Growth Model. Plant Physiol. Biochem. 2010, 48, 496–505, doi:10.1016/j.plaphy.2010.01.019.
7. Serafini-Fracassini, D.; Del Duca, S.; D'Orazi, D. First Evidence for Polyamine Conjugation Mediated by an Enzymic Activity in Plants. Plant Physiology 1988, 87, 757–761, doi:10.1104/pp.87.3.757.
8. Serafini-Fracassini, D.; Del Duca, S.; D'Orazi, D.; Mossetti, U. Conjugation of polyamines by an enzyme activity in dividing cells of *Helianthus tuberosus*. In Perspectives in polyamine research.; Wichtig editore, 1988; pp. 89–92 ISBN 88-85053-36 X.
9. Serafini-Fracassini, D.; Del Duca, S.; Beninati, S. Plant Transglutaminases. Phytochemistry 1995, 40, 355–365, doi:10.1016/0031-9422(95)00243-Z.
10. Del Duca, S.; Beninati, S.; Serafini-Fracassini, D. Polyamines in Chloroplasts: Identification of Their Glutamyl and Acetyl Derivatives. Biochemical Journal 1995, 305, 233–237, doi:10.1042/bj3050233.
11. Serafini-Fracassini, D.; Del Duca, S. Transglutaminases: Widespread Cross-Linking Enzymes in Plants. Annals of Botany 2008, 102, 145–152, doi:10.1093/aob/mcn075.
12. Aloisi, I.; Cai, G.; Serafini-Fracassini, D.; Del Duca, S. Polyamines in Pollen: From Microsporogenesis to Fertilization. Front. Plant Sci. 2016, 7, doi:10.3389/fpls.2016.00155.
13. Parrotta, L.; Tanwar, U.K.; Aloisi, I.; Sobieszczuk-Nowicka, E.; Arasimowicz-Jelonek, M.; Del Duca, S. Plant Transglutaminases: New Insights in Biochemistry, Genetics, and Physiology. Cells 2022, 11, doi:10.3390/cells11091529.
14. Bagni, N. Polyamines in plant growth and development. In The Physiology of Polyamines, Vol. II; Bachrach U. and Heimer Y.M., Eds.: Boca raton, Florida, 1989; Vol. II, pp. 107–120.
15. Bagni, N.; Serafini-Fracassini, D. The role of polyamines as growth factors in higher plants and their mechanism of action. In Proceedings of the Proceedings of Conference on Plant Growth Substances; Hirokawa Publishing Company: Tokyo, 1974; pp. 1205–1217.
16. Del Duca, S.; Bonner, P.L.R.; Aloisi, I.; Serafini-Fracassini, D.; Cai, G. Determination of Transglutaminase Activity in Plants. In Methods in Molecular Biology; 2018; Vol. 1694, pp. 173–200.
17. D'Orazi, D.; Serafini-Fracassini, D.; Bagni, N. Polyamine Effects on the Stability of DNA-Actinomycin D Complex. Biochemical and Biophysical Research Communications 1979, 90, 362–367, doi:10.1016/0006-291X(79)91633-4.

18. Bagni, N.; Corsini, E.; Serafini-Fracassini, D.S. Growth-Factors and Nucleic Acid Synthesis in *Helianthus tuberosus* I. Reversal of Actinomycin D Inhibition by Spermidine. Physiologia Plantarum 1971, 24, 112–117, doi:10.1111/j.1399-3054.1971.tb06727.x
19. Serafini-Fracassini, D.; Torrigiani, P.; Branca, C. Polyamines Bound to Nucleic Acids during Dormancy and Activation of Tuber Cells of *Helianthus tuberosus*. Physiologia Plantarum 1984, 60, 351–357, doi:10.1111/j.1399-3054.1984.tb06075.x.
20. Tassoni, A.; Antognoni, F.; Bagni, N. Polyamine Binding to Plasma Membrane Vesicles Isolated from Zucchini Hypocotyls. Plant Physiology 1996, 110, 817–824, doi:10.1104/pp.110.3.817.
21. D'Orazi, D.; Bagni, N. *In vitro* Interactions between Polyamines and Pectic Substances. Biochemical and Biophysical Research Communications 1987, 148, 1259–1263, doi:10.1016/S0006-291X(87)80268-1.
22. Serafini-Fracassini, D.; Del Duca, S.; Torrigiani, P. Polyamine Conjugation during the Cell Cycle of *Helianthus tuberosus*: Non Enzymatic and Transglutaminase-like Binding Activity. Plant Physiology and Biochemistry 1989, 27, 659–668.
23. Della Mea, M.; Caparrós-Ruiz, D.; Claparols, I.; Serafini-Fracassini, D.; Rigau, J. AtPng1p. The First Plant Transglutaminase. Plant Physiology 2004, 135, 2046–2054, doi:10.1104/pp.104.042549.
24. Della Mea, M.; Di Sandro, A.; Dondini, L.; Del Duca, S.; Vantini, F.; Bergamini, C.; Bassi, R.; Serafini-Fracassini, D. A Zea Mays 39-KDa Thylakoid Transglutaminase Catalyses the Modification by Polyamines of Light-Harvesting Complex II in a Light-Dependent Way. Planta 2004, 219, 754–764, doi:10.1007/s00425-004-1278-6.
25. Beninati, S.; Iorio, R.A.; Tasco, G.; Serafini-Fracassini, D.; Casadio, R.; Del Duca, S. Expression of Different Forms of Transglutaminases by Immature Cells of *Helianthus tuberosus* Sprout Apices. Amino Acids 2013, 44, 271–283, doi:10.1007/s00726-012-1411-y.
26. Del Duca, S.; Serafini-Fracassini, D. Transglutaminases of Higher, Lower Plants and Fungi. Progress in Experimental Tumor Research 2005, 38, 223–247
27. Del Duca, S.; Dondini, L.; Della Mea, M.; Munoz De Rueda, P.; Serafini-Fracassini, D. Factors Affecting Transglutaminase Activity Catalysing Polyamine Conjugation to Endogenous Substrates in the Entire Chloroplast. Plant Physiology and Biochemistry 2000, 38, 429–439, doi:10.1016/S0981-9428(00)00761-0.
28. Del Duca, S.; Tidu, V.; Bassi, R.; Esposito, C.; Serafmi-Fracassini, D. Identification of Chlorophyll-a/b Proteins as Substrates of Transglutaminase Activity in Isolated Chloroplasts of *Helianthus tuberosus* L. Planta 1994, 193, 283–289, doi:10.1007/BF00192542.
29. Del Duca, S.; Faleri, C.; Iorio, R.A.; Cresti, M.; Serafini-Fracassini, D.; Cai, G. Distribution of Transglutaminase in Pear Pollen Tubes in Relation to Cytoskeleton and Membrane Dynamics. Plant Physiology 2013, 161, 1706–1721, doi:10.1104/pp.112.212225.
30. Del Duca, S.; Bregoli, A.M.; Bergamini, C.; Serafini-Fracassini, D. Transglutaminase-Catalyzed Modification of Cytoskeletal Proteins by Polyamines during the Germination of *Malus domestica* Pollen. Sexual Plant Reproduction 1997, 10, 89–95, doi:10.1007/s004970050072.
31. Del Duca, S.; Serafini-Fracassini, D.; Bonner, P.L.R.; Cresti, M.; Cai, G. Effects of Post-Translational Modifications Catalysed by Pollen Transglutaminase on the Functional Properties of Microtubules and Actin Filaments. Biochemical Journal 2009, 418, 651–664, doi:10.1042/BJ20081781.
32. Della Mea, M.; Serafini-Fracassini, D.; Del Duca, S. Programmed Cell Death: Similarities and Differences in Animals and Plants. A Flower Paradigm. Amino Acids 2007, 33, 395–404, doi:10.1007/s00726-007-0530-3.
33. Bachrach, U. The Early History of Polyamine Research. Plant Physiology and Biochemistry 2010, 48, 490–495, doi:10.1016/j.plaphy.2010.02.003.
34. Rosenheim, O. The isolation of spermine phosphate from semen and testis. Biochem J. 1924, 18, 1253–1263.

35. Morgan, D.M.L. Polyamines: An Overview. Applied Biochemistry and Biotechnology - Part B Molecular Biotechnology 1999, 11, 229–250, doi:10.1007/BF02788682.
36. Schuber, F. Influence of Polyamines on Membrane Functions. Biochemical Journal 1989, 260, 1–10, doi:10.1042/bj2600001.
37. Bagni, N.; Creus, J.; Pistocchi, R. Distribution of Cadaverine and Lysine Decarboxylase Activity in Nicotiana Glauca Plants. Journal of Plant Physiology 1986, 125, 9–15, doi:10.1016/S0176-1617(86)80238-3.
38. Vera-Sirera, F.; Minguet, E.G.; Singh, S.K.; Ljung, K.; Tuominen, H.; Blázquez, M.A.; Carbonell, J. Role of Polyamines in Plant Vascular Development. Plant Physiology and Biochemistry 2010, 48, 534–539, doi:https://doi.org/10.1016/j.plaphy.2010.01.011.
39. Marina, M.; Vera-Sirera, F.V.; Rambla, J.L.; Gonzalez, M.E.; Blázquez, M.A.; Carbonell, J.; Pieckenstain, F.L.; Ruiz, O.A. Thermospermine Catabolism Increases Arabidopsis Thaliana Resistance to Pseudomonas Viridiflava. Journal of Experimental Botany 2013, 64, 1393–1402, doi:10.1093/jxb/ert012.
40. Bagni, N.; Tassoni, A. Biosynthesis, Oxidation and Conjugation of Aliphatic Polyamines in Higher Plants. Amino Acids 2001, 20, 301–317, doi:10.1007/s007260170046.
41. Torrigiani, P.; Serafini-Fracassini, D.; Biondi, S.; Bagni, N. Evidence for the Subcellular Localization of Polyamines and Their Biosynthetic Enzymes in Plant Cells. Journal of Plant Physiology 1986, 124, 23–29, doi:10.1016/S0176-1617(86)80174-2.
42. Kakkar, R.K.; Nagar, P.K. Distribution and Changes in Endogenous Polyamines during Winter Dormancy in Tea [Camellia sinensis L. (O) Kuntze]. Journal of Plant Physiology 1997, 151, 63–67, doi:10.1016/S0176-1617(97)80037-5.
43. Tiburcio, A.F.; Altabella, T.; Bitrián, M.; Alcázar, R. The Roles of Polyamines during the Lifespan of Plants: From Development to Stress. Planta 2014, 240, 1–18, doi:10.1007/s00425-014-2055-9.
44. Pistocchi, R.; Keller, F.; Bagni, N.; Matile, P. Transport and Subcellular Localization of Polyamines in Carrot Protoplasts and Vacuoles. Plant Physiology 1988, 87, 514–518, doi:10.1104/pp.87.2.514.
45. Antognoni, F.; Casali, P.; Pistocchi, R.; Bagni, N. Kinetics and Calcium-Specificity of Polyamine Uptake in Carrot Protoplasts. Amino Acids 1994, 6, 301–309, doi:10.1007/BF00813750.
46. 24. Pistocchi, R.; Antognoni, F.; Bagni, N.; Zannoni, D. Spermidine Uptake by Mitochondria of *Helianthus tuberosus*. Plant Physiology 1990, 92, 690–695, doi:10.1104/pp.92.3.690.
47. Fujita, M.; Shinozaki, K. Polyamine Transport Systems in Plants. In Polyamines: A Universal Molecular Nexus for Growth, Survival, and Specialized Metabolism; Kusano, T., Suzuki, H., Eds.; Springer Japan: Tokyo, 2015; pp. 179–185 ISBN 978-4-431-55212-3.
48. Caffaro, S.V.; Antognoni, F.; Scaramagli, S.; Bagni, N. Polyamine Translocation Following Photoperiodic Flowering Induction in Soybean. Physiologia Plantarum 1994, 91, 251–256, doi:10.1111/j.1399-3054.1994.tb00426.x
49. Pegg, A.E. Introduction to the Thematic Minireview Series: Sixty plus Years of Polyamine Research. Journal of Biological Chemistry 2018, 293, 18681–18692, doi:10.1074/jbc.TM118.006291.
50. Rabiti, A.L.; Pistocchi, R.; Bagni, N. Putrescine Uptake and Translocation in Higher Plants. Physiologia Plantarum 1989, 77, 225–230, doi:10.1111/j.1399-3054.1989.tb04973.x.
51. Antognoni, F.; Fornalè, S.; Grimmer, C.; Komor, E.; Bagni, N. Long-Distance Translocation of Polyamines in Phloem and Xylem of Ricinus Communis L. Plants. Planta 1998, 204, 520–527, doi:10.1007/s004250050287.
52. Biasi, R.; Bagni, N.; Costa, G. Endogenous Polyamines in Apple and Their Relationship to Fruit Set and Fruit Growth. Physiologia Plantarum 1988, 73, 201–205, doi:10.1111/j.1399-3054.1988.tb00586.x.

53. Kotzabasis, K.; Fotinou, C.; Roubelakis-Angelakis, K.A.; Ghanotakis, D. Polyamines in the Photosynthetic Apparatus - Photosystem II Highly Resolved Subcomplexes Are Enriched in Spermine. Photosynthesis Research 1993, 38, 83–88, doi:10.1007/BF00015064.
54. Bagni, N.; Barbieri, P.; Torrigiani, P. Polyamine Titer and Biosynthetic Enzymes during Tuber Formation of *Helianthus tuberosus*. Journal of Plant Growth Regulation 1983, 2, 177–184, doi:10.1007/BF02042246.
55. D'Orazi D. and Bagni N. (1987). Ornithine decarboxylase activity in *Helianthus tuberosus*. Physiol. Plant., 71, 177-183
56. Bagni, N.; Calzoni, G.L.; Speranza, A. Polyamines as Sole Nitrogen Sources for *Helianthus tuberosus* Explants *In vitro*. New Phytologist 1978, 80, 317–323, doi:10.1111/j.1469-8137.1978.tb01564.x.
57. Smith, T.A.; Bagni, N.; Serafini-Fracassini, D. The formation of amines and their derivatives in plants. In Nitrogen Assimilitation of Plants; Hewithh E.J., Academic Press, 1979; pp. 557–562
58. Bernet, E.; Claparols, I.; Dondini, L.; Santos, M.; Serafini-Fracassini, D.; Torné, J.M. Changes in Polyamine Content, Arginine and Ornithine Decarboxylases and Transglutaminase Activities during Light/Dark Phases (of Initial Differentiation) in Maize Calluses and Their Chloroplasts. Plant Physiology and Biochemistry 1999, 37, 899–909, doi:10.1016/S0981-9428(99)00104-7.
59. Kuznetsov, Vl.V.; Radyukina, N.L.; Shevyakova, N.I. Polyamines and Stress: Biological Role, Metabolism, and Regulation. Russian Journal of Plant Physiology 2006, 53, 583–604, doi:10.1134/S1021443706050025.
60. Tyagi, A.; Ali, S.; Ramakrishna, G.; Singh, A.; Park, S.; Mahmoudi, H.; Bae, H. Revisiting the Role of Polyamines in Plant Growth and Abiotic Stress Resilience: Mechanisms, Crosstalk, and Future Perspectives. Journal of Plant Growth Regulation 2023, 42, 5074–5098, doi:10.1007/s00344-022-10847-3.
61. Hasan, M.M.; Skalicky, M.; Jahan, M.S.; Hossain, M.N.; Anwar, Z.; Nie, Z.; Alabdallah, N.M.; Brestic, M.; Hejnak, V.; Fang, X.-W. Spermine: Its Emerging Role in Regulating Drought Stress Responses in Plants. Cells 2021, 10, 1–15, doi:10.3390/cells10020261.
62. Del Duca, S.; Creus, J.A.; D'Orazi, D.; Dondini, L.; Bregoli, A.M.; Serafini-Fracassini, D. Tuber Vegetative Stages and Cell Cycle in *Helianthus tuberosus*: Protein Pattern and Their Modification by Spermidine. Journal of Plant Physiology 2000, 156, 17–25, doi:10.1016/S0176-1617(00)80267-9.
63. Bagni, N.; Torrigiani, P.; Barbieri, P. Effect of Various Inhibitors of Polyamine Synthesis on the Growth of *Helianthus tuberosus*. Medical Biology 1981, 59, 403–409.
64. Bagni, N.; Pistocchi, R. Putrescine Uptake in Saintpaulia Petals. Plant Physiology 1985, 77, 398–402, doi:10.1104/pp.77.2.398.
65. Bagni, N.; Serafini-Fracassini, D. The role of polyamines as growth factors in higher plants and their mechanism of action. In Proceedings of the Proceedings of Conference on Plant Growth Substances; Hirokawa Publishing Company: Tokyo, 1974; pp. 1205–1217.
66. Del Duca, S.; Favali, M.A.; Serafini-Fracassini, D.; Pedrazzini, R. Transglutaminase Activity during Greening and Growth of *Helianthus tuberosus* Explants in Vitro. Protoplasma 1993, 174, 1–9, doi:10.1007/BF01404036.
67. Aloisi, I.; Cai, G.; Tumiatti, V.; Minarini, A.; Del Duca, S. Natural Polyamines and Synthetic Analogs Modify the Growth and the Morphology of Pyrus Communis Pollen Tubes Affecting ROS Levels and Causing Cell Death. Plant Science 2015, 239, 92–105, doi:10.1016/j.plantsci.2015.07.008.
68. Gentile, A.; Antognoni, F.; Iorio, R.A.; Distefano, G.; Casas, G.L.; La Malfa, S.; Serafini-Fracassini, D.; Del Duca, S. Polyamines and Transglutaminase Activity Are Involved in Compatible and Self-Incompatible Pollination of Citrus Grandis. Amino Acids 2012, 42, 1025–1035, doi:10.1007/s00726-011-1017-9.
69. Serafini-Fracassini, D.; Della Mea, M.; Parrotta, L.; Faleri, C.; Cai, G.; Del Duca, S.; Aloisi, I. AtPng1 Knockout Mutant of Arabidopsis Thaliana Shows a Juvenile Phenotype, Morpho-

Functional Changes, Altered Stress Response and Cell Wall Modifications. Plant Physiology and Biochemistry 2021, 167, 11–21, doi:10.1016/j.plaphy.2021.07.024.
70. Cai, G.; Della Mea, M.; Faleri, C.; Fattorini, L.; Aloisi, I.; Serafini-Fracassini, D.; Del Duca, S. Spermine Either Delays or Promotes Cell Death in *Nicotiana tabacum* L. Corolla Depending on the Floral Developmental Stage and Affects the Distribution of Transglutaminase. Plant Science 2015, 241, 11–22, doi:10.1016/j.plantsci.2015.09.023.
71. Handa, A.K.; Mattoo, A.K. Differential and Functional Interactions Emphasize the Multiple Roles of Polyamines in Plants. Plant Physiology and Biochemistry 2010, 48, 540–546, doi:10.1016/j.plaphy.2010.02.009.
72. Sánchez-Jiménez, F.; Medina, M.Á.; Villalobos-Rueda, L.; Urdiales, J.L. Polyamines in Mammalian Pathophysiology. Cellular and Molecular Life Sciences 2019, 76, 3987–4008, doi:10.1007/s00018-019-03196-0.
73. Batista De Carvalho, A.L.M.; Mamede, A.P.; Dopplapudi, A.; Garcia Sakai, V.; Doherty, J.; Frogley, M.; Cinque, G.; Gardner, P.; Gianolio, D.; Batista De Carvalho, L.A.E.; et al. Anticancer Drug Impact on DNA-a Study by Neutron Spectroscopy Coupled with Synchrotron-Based FTIR and EXAFS. Physical Chemistry Chemical Physics 2019, 21, 4162–4175, doi:10.1039/c8cp05881d.
74. Del Duca, S.; Serafini-Fracassini, D.; Cai, G. Senescence and Programmed Cell Death in Plants: Polyamine Action Mediated by Transglutaminase. Front. Plant Sci. 2014, 5, doi:10.3389/fpls.2014.00120.
75. Lorand, L.; Graham, R.M. Transglutaminases: Crosslinking Enzymes with Pleiotropic Functions. Nature Reviews Molecular Cell Biology 2003, 4, 140–156, doi:10.1038/nrm1014
76. Mattoo, A.K.; Upadhyay, R.K.; Rudrabhatla, S. Abiotic Stress in Crops: Candidate Genes, Osmolytes, Polyamines, and Biotechnological Intervention. In Elucidation of Abiotic Stress Signaling in Plants: Functional Genomics Perspectives, Volume 2; 2015; pp. 415–437.
77. Igarashi, K.; Kashiwagi, K. Characteristics of Cellular Polyamine Transport in Prokaryotes and Eukaryotes. Plant Physiology and Biochemistry 2010, 48, 506–512, doi:10.1016/j.plaphy.2010.01.017.
78. Mossetti, U.; Serafini Fracassini, D.; Del Duca, S.; D'Orazi, D. Conjugated polyamines during dormancy and activation of tuber of Jerusalem artichoke. In Proceedings of the Proceedings of the International Symposium Conjugated Plant Hormones, Structure, Metabolism and Function; K. Schreiber, H.R. Schutte, G. Sembnder: Berlino, 1987; Vol. 1, pp. 369–375.
79. Bachrach, U. Naturally Occurring Polyamines: Interaction with Macromolecules. Current Protein and Peptide Science 2005, 6, 559–566, doi:10.2174/138920305774933240.
80. Cocucci, S.; Bagni, N. Polyamine-Induced Activation of Protein Synthesis in Ribosomal Preparation from *Helianthus tuberosus* Tissue. Life Sciences 1968, 7, 113–120, doi:10.1016/0024-3205(68)90294-4.
81. Belda-Palazón, B.; Ruiz, L.; Martí, E.; Tárraga, S.; Tiburcio, A.F.; Culiáñez, F.; Farràs, R.; Carrasco, P.; Ferrando, A. Aminopropyltransferases Involved in Polyamine Biosynthesis Localize Preferentially in the Nucleus of Plant Cells. PLoS ONE 2012, 7, doi:10.1371/journal.pone.0046907.
82. Sato, N.; Ohtake, Y.; Kato, H.; Abe, S.; Kohno, H.; Ohkubo, Y. Effects of Polyamines on Histone Polymerization. Journal of Protein Chemistry 2003, 22, 303–307, doi:10.1023/A:1025032906494.
83. Cohen, S.S. A Guide to the Polyamines; Oxford University Press.; Cohen, S.S.: New York, 1998; ISBN 0-19-511064-1.
84. Schuber, F. Influence of Polyamines on Membrane Functions. Biochemical Journal 1989, 260, 1–10, doi:10.1042/bj2600001.
85. Koenig, H.; Goldstone, A.; Lu, C.Y. Polyamines Regulate Calcium Fluxes in a Rapid Plasma Membrane Response. Nature 1983, 305, 530–534, doi:10.1038/305530a0.

86. Mariani, P.; D'Orazi, D.; Bagni, N. Polyamines in Primary Walls of Carrot Cells: Endogenous Content and Interactions. Journal of Plant Physiology 1989, 135, 508–510, doi:10.1016/S0176-1617(89)80113-0.
87. Berta, G.; Altamura, M.M.; Fusconi, A.; Cerruti, F.; Capitani, F.; Bagni, N. The Plant Cell Wall Is Altered by Inhibition of Polyamine Biosynthesis. New Phytologist 1997, 137, 569–577, doi:10.1046/j.1469-8137.1997.00868.x.
88. Bassard, J.-E.; Ullmann, P.; Bernier, F.; Werck-Reichhart, D. Phenolamides: Bridging Polyamines to the Phenolic Metabolism. Phytochemistry 2010, 71, 1808–1824, doi:10.1016/j.phytochem.2010.08.003.
89. Lütz, C.; Navakoudis, E.; Seidlitz, H.K.; Kotzabasis, K. Simulated Solar Irradiation with Enhanced UV-B Adjust Plastid- and Thylakoid-Associated Polyamine Changes for UV-B Protection. Biochimica et Biophysica Acta - Bioenergetics 2005, 1710, 24–33, doi:10.1016/j.bbabio.2005.09.001.
90. Michael, A.J. Biosynthesis of Polyamines and Polyamine-Containing Molecules. Biochemical Journal 2016, 473, 2315–2329, doi:10.1042/BCJ20160185.
91. Grienenberger, E.; Besseau, S.; Geoffroy, P.; Debayle, D.; Heintz, D.; Lapierre, C.; Pollet, B.; Heitz, T.; Legrand, M. A BAHD Acyltransferase Is Expressed in the Tapetum of Arabidopsis Anthers and Is Involved in the Synthesis of Hydroxycinnamoyl Spermidines. Plant Journal 2009, 58, 246–259, doi:10.1111/j.1365-313X.2008.03773.x.
92. Martin-Tanguy, J. Metabolism and Function of Polyamines in Plants: Recent Development (New Approaches). Plant Growth Regulation 2001, 34, 135–148, doi:10.1023/A:1013343106574.
93. Esposito, C.; Caputo, I. Mammalian Transglutaminases: Identification of Substrates as a Key to Physiological Function and Physiopathological Relevance. FEBS Journal 2005, 272, 615–631, doi:10.1111/j.1742-4658.2004.04476.x.
94. Icekson, I.; Apelbaum, A. Evidence for Transglutaminase Activity in Plant Tissue. Plant Physiology 1987, 84, 972–974, doi:10.1104/pp.84.4.972.
95. Della Mea, M.; Caparrós-Ruiz, D.; Claparols, I.; Serafini-Fracassini, D.; Rigau, J. AtPng1p. The First Plant Transglutaminase. Plant Physiology 2004, 135, 2046–2054, doi:10.1104/pp.104.042549.
96. Falcone, P.; Serafini-Fracassini, D.; Del Duca, S. Comparative Studies of Transglutaminase Activity and Substrates in Different Organs of *Helianthus tuberosus*. Journal of Plant Physiology 1993, 142, 265–273, doi:10.1016/S0176-1617(11)80421-9.
97. Sobieszczuk-Nowicka, E.; Legocka, J. Plastid-Associated Polyamines: Their Role in Differentiation, Structure, Functioning, Stress Response and Senescence. Plant Biology 2014, 16, 297–305, doi:10.1111/plb.12058.
98. Ioannidis, N.E.; Kotzabasis, K. Polyamines in Chemiosmosis *In vivo*: A Cunning Mechanism for the Regulation of ATP Synthesis during Growth and Stress. Frontiers in Plant Science 2014, 5, doi:10.3389/fpls.2014.00071.
99. Pál, M.; Szalai, G.; Gondor, O.K.; Janda, T. Unfinished Story of Polyamines: Role of Conjugation, Transport and Light-Related Regulation in the Polyamine Metabolism in Plants. Plant Science 2021, 308, doi:10.1016/j.plantsci.2021.110923.
100. Dondini, L.; Bonazzi, S.; Del Duca, S.; Bregoli, A.M.; Serafini-Fracassini, D. Acclimation of Chloroplast Transglutaminase to High NaCI Concentration in a Polyamine-Deficient Variant Strain of Dunaliella Salina and in Its Wild Type. Journal of Plant Physiology 2001, 158, 185–197, doi:10.1078/0176-1617-00099.
101. Das, K.C.; Misra, H.P. Hydroxyl Radical Scavenging and Singlet Oxygen Quenching Properties of Polyamines. Molecular and Cellular Biochemistry 2004, 262, 127–133, doi:10.1023/B:MCBI.0000038227.91813.79.
102. Dondini, L.; Del Duca, S.; Dall'Agata, L.; Bassi, R.; Gastaldelli, M.; Della Mea, M.; Di Sandro, A.; Claparols, I.; Serafini-Fracassini, D. on *Helianthus tuberosus* Chloroplast Transglutaminases and Their Substrates. Planta 2003, 217, 84–95, doi:10.1007/s00425-003-0998-3.

103. Yasumoto, K.; Sakata, T.; Yasumoto, J.; Yasumoto-Hirose, M.; Sato, S.-I.; Mori-Yasumoto, K.; Jimbo, M.; Kusumi, T.; Watabe, S. Atmospheric CO2 Captured by Biogenic Polyamines Is Transferred as a Possible Substrate to Rubisco for the Carboxylation Reaction. Scientific Reports 2018, 8, doi:10.1038/s41598-018-35641-8.
104. Di Sandro, A.; Del Duca, S.; Verderio, E.; Hargreaves, A.J.; Scarpellini, A.; Cai, G.; Cresti, M.; Faleri, C.; Iorio, R.A.; Hirose, S.; et al. An Extracellular Transglutaminase Is Required for Apple Pollen Tube Growth. Biochemical Journal 2010, 429, 261–271, doi:10.1042/BJ20100291.
105. Aloisi, I.; Piccini, C.; Cai, G.; Del Duca, S. Male Fertility under Environmental Stress: Do Polyamines Act as Pollen Tube Growth Protectants? International Journal of Molecular Sciences 2022, 23, doi:10.3390/ijms23031874.
106. Iorio, R.A.; Di Sandro, A.; Paris, R.; Pagliarani, G.; Tartarini, S.; Ricci, G.; Serafini-Fracassini, D.; Verderio, E.; Del Duca, S. Simulated Environmental Criticalities Affect Transglutaminase of Malus and Corylus Pollens Having Different Allergenic Potential. Amino Acids 2012, 42, 1007–1024, doi:10.1007/s00726-011-1043-7.
